# Anti-toxoplasma Antibody Prevalence and Cost-effectiveness in Pregnant Women at the King Abdulaziz University Hospital, Jeddah, Saudi Arabia

**DOI:** 10.7759/cureus.6675

**Published:** 2020-01-16

**Authors:** Ashwag H Mohajab, Hisham Z Alshehri, Riyadh O Shati, Ahmed A Alshehri, Mohammed A Alafghani, Abdulrahman Alasmari, Maan Almahi, Ayman Oraif

**Affiliations:** 1 Medicine, Jazan University, Jazan, SAU; 2 Medicine, King Abdulaziz University, Jeddah, SAU; 3 Orthopaedics, Jeddah University, Jeddah, SAU; 4 Anesthesiology, King Abdulaziz University, Jeddah, SAU; 5 Obstetrics and Gynecology, King Abdulaziz University, Jeddah, SAU

**Keywords:** toxoplasma gondii, pregnant women, anti-toxoplasmosis antibodies, parasites

## Abstract

Background: Toxoplasma gondii (T. gondii) is one of the most prevalent infectious parasites in humans worldwide. The diagnosis of toxoplasmosis is based on serological screening through the detection of anti-toxoplasmosis antibodies: IgG, which indicates previous exposure and the presence of active immunity, and IgM, which indicates a recent infection. We aimed to determine the prevalence of anti-toxoplasma antibodies in pregnant women at King Abdulaziz University Hospital (KAUH), Jeddah, Saudi Arabia, and explore the cost-effectiveness of anti-toxoplasma screening.

Method: This was an analytic retrospective study of women who underwent serology testing for anti-toxoplasmosis antibodies at KAUH in 2013-2018. Data were collected from hospital documentation and IBM Statistical Package for Social Sciences (SPSS Inc., Chicago, IL) version 22 was used for analysis.

Result: Of the 9,098 pregnant women seen at KAUH, 2,754 had undergone the test, and 38 had a positive result, i.e., a seroprevalence rate of 1.4%. Most women were Saudis (57.9%), and almost all were multiparous. Of those with a positive result, 36.8% were in the third trimester. Most births were by spontaneous vaginal delivery (65.8%). Twelve (31.6%) of the women with toxoplasmosis experienced obstetric complications. The estimated total cost of screening the pregnancies was US $919,646.00

Conclusion: The prevalence of pregnant women with a positive anti-toxoplasmosis test result was low, and we believe there is no net benefit from screening all pregnant women for toxoplasmosis. Primary prevention should be through health education, and we recommend screening only women with high-risk pregnancies.

## Introduction

Toxoplasma gondii (T.gondii) is a common intracellular parasite that is considered one of the most prevalent infectious parasites in humans worldwide [1]. Transmission primarily occurs through contact with cats; other routes include consumption of contaminated water and food, including raw meat and vegetables, and through soil [2]. In some patients, and especially immunocompromised patients and neonates with congenitally acquired infections, T. gondii can cause severe complications and morbidity [3].

In pregnancy, the most significant risk occurs during the first trimester; even though the transmission rate of T. gondii is low, the related morbidity is high during this period, and it can result in severe congenital anomalies [4]. This is in contrast to the third trimester, which is characterized by a high transmission rate but low morbidity [4]. Some mothers who acquire the infection several months before conception are shown to have a low risk of congenital toxoplasmosis [5]. Transplacental transmission results in congenital toxoplasmosis, with a broad spectrum of inherent complications, which can vary according to the trimester in which the parasitic infection was transmitted [5]. Stillbirth and spontaneous abortion are common in the first trimester. Some of the affected infants may not develop signs and maybe asymptomatic [6]; however, years later, most will develop neurological problems such as seizures, microcephaly, hydrocephalus, mental retardation, hearing loss, and retinochoroiditis with or without blindness [7].

The diagnosis of toxoplasmosis is based on serological screening through the detection of anti-toxoplasmosis antibodies; IgG, which indicates previous exposure and the presence of active immunity, and IgM, which indicates a recent infection [8].

In Riyadh, Saudi Arabia, a study conducted in 2015, explored the prevalence of toxoplasmosis among 250 pregnant women from multicenter healthcare and community-based settings. It estimated the seropositivity of anti-toxoplasma IgG and IgM antibodies to be 32% and 6.4%, respectively [9]. A study conducted in Najran Province, Saudi found anti-toxoplasma IgG and IgM antibodies in 20 out of 96 pregnant women (20.8%) attending special prenatal clinics from 2012 to 2013 [10]. Similarly, a study conducted in Al-Ahsa, Saudi on the seroprevalence and possible risk factors among 680 pregnant women attending a maternity hospital found 8.8% to be seropositive for anti-T.gondii (IgM) [11]. There was a substantially higher result for anti-T.gondii IgG, at 51.4%, [11] which is higher than a previous value of 35.6% reported in 2002 in Makkah Province, Saudi [12].

It has previously been observed that prenatal screening resulted in substantial cost savings due to a reduction in congenital toxoplasmosis in Austria, with a total annual saving of US $2.1 million [13]. This study also showed that prenatal screening and treatment has a considerable cost-saving impact on the Austrian national economy, and that the screening program saved Austrian society about €448 million in costs for the birth cohorts from 1992 to 2008 [13]. This concept has recently been challenged by false-positive test results being reflected in these crude net cost estimates and information on treatment effectiveness.

In contrast, neonatal screening can be added to established Guthrie card-based testing, and is estimated to cost between £1.85 million and £3.5 million for a population of 700,000 live births for each infected child prevented, most of whom would be asymptomatic [14]. However, limited studies have been conducted to explore the prevalence and cost-effectiveness of the screening test for T. Gondii infection among Saudi pregnant women. King Abdulaziz University Hospital (KAUH), a tertiary and educational hospital with more than 800 beds, located in the western region of Saudi, in Jeddah city, has implemented prenatal screening for toxoplasmosis. However, a major drawback is that limited studies have been conducted to determine the anti-toxoplasma antibody prevalence in KAUH. Therefore, this study aims not only to show the incidence of anti-toxoplasma antibody in pregnant women at KAUH, but also to find out whether its anti-toxoplasma testing is cost-effective.

## Materials and methods

Our study is retrospective and analyses laboratory data on T.gondii screening carried out at KAUH from January 2013 to February 2018. The study group comprised all pregnant women who attended the antenatal care unit and were screened using the anti-toxoplasmosis antibodies serology test. A total of 9,094 women delivered their babies at KAUH during the study period, but pregnant women who had not undergone the test were excluded.

After identifying a positive result, our aim was to look for any complications in the mother or their fetus. Samples from all pregnant women who underwent prenatal screening were tested for anti-toxoplasma antibodies using enzyme-linked immunosorbent assay scanning for IgM and IgG. The data were obtained from the electronic medical documentation and registration system (Phoenix, Phoenix Business Solutions). A structured data sheet was used to collect the nationality, maternal age (years), gestational age (GA) at delivery, gravidity, parity, anti-toxoplasmosis level, and GA at which the test was performed. In those with a positive test result the history of complications was examined, which included oligohydramnios, anhydramnios, cardiomegaly, intrauterine growth restriction (IUGR), abortion, fetal hydrops, premature rupture of membranes (PROM) and intrauterine fetal demise (IUFD). The IBM Statistical Package for Social Sciences (SPSS Inc., Chicago, IL) version 22, was used to analyze the data. The study protocol was approved by the Institutional Review Board of our university hospital.

## Results

Of the 9,098 pregnant women attending the antenatal care unit, 2,754 had taken the test, and only 38 were found to be positive - a seroprevalence of 1.4%. Most of these pregnant women were Saudis (57.9%), and almost all were multiparous, with just one woman being primiparous, as shown in Table 1. Among the 38 pregnant women who tested positive, 71.1% were in the age group 29-38 years, as shown in Table 1. The mean age was 32 years. Of those who tested positive for anti-toxoplasma IgG antibody, 28.9% were in the first trimester, 34.2% in the second and 36.8% in the third trimester (Table 1). Mean GA at delivery was 37 weeks.

**Table 1:** Delivery and data on toxoplasmosis in the 38 pregnant women that tested positive SVD: spontaneous vaginal delivery; CS: cesarean section; GA: gestational age.

Variable	Mean ± SD or n (%)
Type of delivery	
SVD	25 (65.8%)
CS	13 (34.2%)
GA at delivery (wks)	37.35 ± 3.11
Anti-toxoplasmosis level (IU/mL)	284.62 ± 249.40
GA at which the test was done (wks)	22.63 ± 11.21

Most of the births were by spontaneous vaginal delivery (65.8%), as shown in Table 2. Twelve of the 38 women (31.6%) who tested positive for toxoplasmosis had obstetric complications: four pregnancies (10.6%) were complicated by oligohydramnios and anhydramnios, three (7.9%) ended up with IUGR and IUFD, two (5.3%) were complicated by fetal hydrops and PROM, and two (5.3%) were complicated by cardiomegaly. Abortion was reported in only one case (2.6%), as shown in Table 3. Of the 12 women with complications, in five cases (13.1%) these were experienced in the first trimester, with a single case (2.6%) in the second trimester, and six cases (15.7%) in the third trimester (Table 3).

**Table 2:** Demographic characteristics of the women who tested positive for anti-toxoplasma antibodies (n = 38)

Variable	Mean ± SD or n (%)
Maternal age (yrs)	32.05 ± 5.54
Gravida	3.32 ± 2.04
Para	2.11 ± 1.56
Nationality	
Saudi	22 (57.9%)
Non-Saudi	16 (42.1%)
Age group (yrs)	
≤ 28	7 (18.4%)
29–38	27 (71.1%)
≥39	4 (10.5%)
Multiparous	37 (97.4%)
Primiparous	1 (2.6%)
Positive result by trimester	
First	11 (28.9%)
Second Third	13 (34.2%) 14 (36.8%)
Complications by trimester	
First	5 (13.1%)
Second	1 (2.6%)
Third	6 (15.7%)

**Table 3:** Association between complications and trimester of Toxoplasma gondii infection (n = 38); values are presented as n (%) IUGR: intrauterine growth restriction; PROM: premature rupture of membranes; IUFD: intrauterine fetal demise.

Variable 1st trimester 2nd trimester 3rd trimester P value Total
Oligohydramnios 1 (2.63) 0 (4.87) 1 (2.63%) 0.564 2 (5.26)
Anhydramnios 1 (2.63) 0 (4.87) 1 (2.63) 0.564 2 (5.26)
Cardiomegaly 0 (5.18) 0 (4.87) 2 (5.26) 0.164 2 (5.26)
IUGR 1 (2.63) 0 (4.87) 1 (2.63) 0.564 2 (5.26)
Abortion 1 (2.63) 0 (4.87) 0 (6.67) 0.284 1 (2.63)
Fetal hydrops 0 (5.18) 0 (4.87) 1 (2.63) 0.415 1 (2.63)
PROM 0 (5.18) 1 (2.63) 0 (6.67) 0.372 1 (2.63)
IUFD 1 (2.63) 0 (4.87) 0 (6.67) 0.284 1 (2.63)
Uncomplicated 6 (15.54) 12 (34.1) 8 (20%) 26 (68.44%)
Complicated 5 (13.1) 1 (2.6) 6 (15.7%) 12 (31.56%)
Total (%) 11 (28.9) 13 (34.2) 14 (36.8%) 38 (100%)

Finally, regarding the related costs, from January 2013 to February 2018, a total of 5,053 tests were carried out for 2,754 pregnancies. The total estimated price of these serological tests was US $919,646.00 (IgG US $93 and IgM US $89).

## Discussion

KAUH has implemented prenatal screening for toxoplasmosis, but limited studies have been conducted to determine the anti-toxoplasma antibody prevalence in pregnant women attending KAUH. Of the 9,094 pregnant women included in this study, 2,754 were screened for anti-toxoplasma IgM and IgG antibodies. Among the tested women, 38 returned a positive result - a seroprevalence of 1.4%. This is very low compared to results reported in other centers of Saudi: 32.5% IgG seroprevalence in Riyadh, [9] 28.5% in Dhahran, [15] 24.1% in Jazan, [16] and 8.57% in Hail [17]. This study aimed to show not only the prevalence of anti-toxoplasma antibodies in pregnant women at KAUH, but also whether its anti-toxoplasma testing program was cost-effective. Our results indicated that from 2013 to 2018, the estimated total cost of 2,754 screened pregnancies was US $919,646.00, which revealed only 38 positive results.

A study conducted in the United Kingdom (UK) showed a lack of evidence and insufficient data on the effectiveness of prenatal and neonatal screening [14]. Also, review of the efficacy of spiramycin showed it to be of no benefit for the treatment of the immunocompetent compared to the immunodeficient. This study estimated the cost of a preventive screening test to be US $932,000, a high price to pay. In comparison, the cost of Down syndrome prenatal screening is remarkably less than that of toxoplasmosis screening, by US $893,000 [14].

A study in Austria [13] showed that early detection would result in early intervention and cost saving. In contrast, a study conducted in the UK [14] showed that prenatal screening and treatment among pregnant women revealed an outcome of fetal complications as inevitable. We assume that instead of doing an annual screening test for T.gondii, health education is considered a primary preventive tool; this will be useful in reducing costs and saving resources. Pregnant women should seek information regarding risks in pregnancy from their physicians; however, it seems that women are not acquiring this critical information about their pregnancy and the risk of diseases.

In this study, 12 (31.6%) of the 38 positive cases were associated with obstetric complications. Two pregnancies were complicated with oligohydramnios and two with IUGR. Other reported complications included two cases of cardiomegaly, which was shown to be associated with T.gondii infection in a case-control study [18]. Moreover, anhydramnios, IUFD, and fetal hydrops occurred in three separate pregnancies, and there was a single case of abortion. First-time exposure to T.gondii may lead to abortion and fetal death; if there a repeat infection by T.gondii, the next pregnancy is less likely to be aborted [14]. The number of complicated cases in this study is relatively higher compared to results obtained from the Polish Mother's Memorial Hospital in Lodz, Poland, where 25.4% of positive anti-T.gondii IgG and IgM pregnancies were associated with obstetric complications [19].

Our study is limited by the fact that the data were obtained from one tertiary hospital and describe the seroprevalence of T.gondii among pregnant women. Hence the results may not be suitable to generalize to the whole community. In addition, seroconversion could not be observed, as only one blood sample from each woman was investigated, with no attempt to confirm such infection using, for example, IgG avidity tests or polymerase chain reaction.

## Conclusions

Our present data revealed that the prevalence of pregnant women who had positive seroprevalence of anti-toxoplasmosis was low, at 1.4%. Hence there is no net benefit from carrying out this screening test for all pregnant women; screening and treatment will not result in any significant changes in fetal health status, and affected infants will be identified during their early life. Based on the results, we believe that it will be beneficial to perform anti-toxoplasma testing only in high-risk pregnancies, instead of routine prenatal screening. Routine testing reveals so few positive cases, for a relatively high price, that it is not cost-effective and wastes resources. Also, by carrying out this test routinely, patients are exposed to unnecessary work-up and investigations. Instead of carrying out these tests for those with non-risk pregnancies, primary prevention by health education should be used. We recommended utilizing the funds currently spent at KAUH on anti-toxoplasma IgG and IgM tests for low-risk pregnancies for other more essential screening tests.
